# Predicting the transmission dynamics of novel coronavirus infection in Shanxi province after the implementation of the “Class B infectious disease Class B management” policy

**DOI:** 10.3389/fpubh.2023.1322430

**Published:** 2023-12-22

**Authors:** Yifei Ma, Shujun Xu, Yuxin Luo, Junlin Peng, Jiaming Guo, Ali Dong, Zhibin Xu, Jiantao Li, Lijian Lei, Lu He, Tong Wang, Hongmei Yu, Jun Xie

**Affiliations:** ^1^School of Public Health, Shanxi Medical University, Taiyuan, China; ^2^Shanxi Center for Disease Control and Prevention, Taiyuan, China; ^3^School of Management, Shanxi Medical University, Taiyuan, China; ^4^Shanxi Provincial Key Laboratory of Major Diseases Risk Assessment, Taiyuan, China; ^5^Department of Biochemistry and Molecular Biology, Shanxi Key Laboratory of Birth Defect and Cell Regeneration, MOE Key Laboratory of Coal Environmental Pathogenicity and Prevention, Shanxi Medical University, Taiyuan, China

**Keywords:** novel coronavirus infection, Class B infectious disease Class B management, liberalization, transmission dynamics, effective reproduction number

## Abstract

**Background:**

China managed coronavirus disease 2019 (COVID-19) with measures against Class B infectious diseases, instead of Class A infectious diseases, in a major shift of its epidemic response policies. We aimed to generate robust information on the transmission dynamics of novel coronavirus infection in Shanxi, a province located in northern China, after the implementation of the “Class B infectious disease Class B management” policy.

**Methods:**

We consolidated infection data in Shanxi province from December 6, 2022 to January 14, 2023 through a network questionnaire survey and sentinel surveillance. A dynamics model of the SEIQHCVR was developed to track the infection curves and effective reproduction number ( $R_{t}$ ).

**Results:**

Our model was effective in estimating the trends of novel coronavirus infection, with the coefficient of determination ( $R^{2}$ ) above 90% in infections, inpatients, and critically ill patients. The number of infections in Shanxi province as well as in urban and rural areas peaked on December 20, 2022, with the peak of inpatients and critically ill patients occurring 2 to 3 weeks after the peak of infections. By the end of January 2023, 87.72% of the Shanxi residents were predicted to be infected, and the outbreak subsequently subsided. A small wave of COVID-19 infections may re-emerge at the end of April. In less than a month, the $R_{t}$ values of positive infections, inpatients and critically ill patients were all below 1.0.

**Conclusion:**

The outbreak in Shanxi province is currently at a low prevalence level. In the face of possible future waves of infection, there is a strong need to strengthen surveillance and early warning.

## Introduction

Currently, coronavirus disease 2019 (COVID-19) continues to be prevalent worldwide, with the Omicron variant becoming the dominant strain (1). In China, people’s lives and health has been the primary consideration in formulating policies for the prevention and control of the epidemic. Based on the understanding of the pathogen, nature and danger of the disease, and immunity of the population and resistance of the health care system, the National Health Commission of the People’s Republic of China issued an announcement to downgrade management of the disease from Class A to Class B in accordance with the country’s law on prevention and treatment of infectious disease and renamed the Chinese term for COVID-19 from “novel coronavirus pneumonia” to “novel coronavirus infection” on January 8, 2023 (2). This is another major shift in its epidemic response policies since January 20, 2020, and was aimed at preventing and controlling COVID-19 in a more scientific and targeted manner and to use resources more effectively to balance epidemic control, prevention and socioeconomic development.

China’s epidemic prevention and control faces a new situation and task, shifting its focus from infection prevention to medical treatment and the prevention of severe cases. The preparation for medical treatment resources is the “first move” and “key move” (3). The “General plan for the implementation of Class B management of novel coronavirus infection” formulated by the State Council’s Joint Prevention and Control Mechanism for New Coronavirus Infection requires the completion of medical resource preparation tasks as soon as possible, focusing on the preparation of inpatient beds and critical care beds, making every effort to protect and treat key populations such as the older adults and children, especially strengthening vaccination and health management for the older adults, and effectively strengthening the prevention and control of the epidemic in rural areas (4).

In terms of the national situation, major cities have passed the peak of infection in late December 2022; however, the treatment of critically ill patients remains in a plateau period. During the Spring Festival travel rush, small- and medium-sized cities and rural areas may usher at the peak of inpatients and critically ill patients. Since the start of the epidemic, the number of novel coronavirus infections in Shanxi province has soared and medical institutions have been operating at a high load. Epidemic prevention, control, and medical treatment are critical periods of maximum strength (5). Shanxi province is close to the “Bohai Sea Economic Circle” centered on Beijing and Tianjin, and is the northernmost of the “Six Central provinces.” Its geographical position, which connects the east and west, makes it an important province in the North China plain. The assessment of the epidemic in Shanxi province can better reflect the development of the epidemic in the surrounding provinces and the entire country. The susceptible-exposed-infected-recovered (SEIR) model is currently a classical transmission dynamics model that can quantify the risk of viral transmission and predict the state of disease occurrence, thereby providing a basis for scientific, accurate, and effective assistance in prevention and control decisions (6, 7). Our study aimed to construct a prediction model in line with the epidemiological characteristics under the policy of “Class B infectious disease Class B management,” so as to dynamically grasp the level and change trend of population infection in a timely manner, and to ensure the smooth transition of the policy adjustment.

## Methods

### Data sources

Shanxi, located at 34°58′–40°72′N, 110°25′–114°55′E, has a population size of 34.91 million. The data on the novel coronavirus infection used in this study were collected through a network questionnaire survey from December 6, 2022 to January 14, 2023. Inclusion criteria: (a) respondents whose place of residence is Shanxi province; (b) respondents who gave informed consent to this survey. Exclusion criteria: (a) although this questionnaire can be filled out by family members instead, questionnaires answered more than five times from the same IP address will be excluded; (b) questionnaires with an answer time of less than 120 s will be excluded. The sample infection ratio calculated from the sample survey was scaled up to the overall population to obtain the daily number of infections in Shanxi province. Data on inpatients and critically ill patients were provided by the Health Commission of Shanxi province through sentinel surveillance.

### Items investigated

The network questionnaire comprised 32 questions on personal information, COVID-19 related prevention, infection, treatment, and mental health status. Sociodemographic characteristics included age, gender, residence, disability, and chronic disease (coronary heart disease, hypertension, diabetes mellitus, cerebrovascular disease, chronic obstructive pulmonary disease, malignant tumor, chronic kidney disease, or others). The part of prevention COVID-19 infection consisted of vaccination status, the time elapsed since last COVID-19 vaccination, and stockpiling of anti-epidemic supplies. Information regarding the time of disease onset from December 6, 2022, and the duration of symptoms related to COVID-19 were investigated. The acute-phase symptoms of COVID-19 include fever, cough, fatigue, nasal obstruction, sore throat, running nose, headaches, myalgia, hypogeusia, eye pain, ostealgia, chills, and vomiting. Treatment was mainly reflected in medications and medical institutions. In aspect of mental health status, anxiety was investigated using the Chinese version of the Generalized Anxiety Disorder scale-7 (GAD-7) at the end of the questionnaire (8). The total GAD-7 score ranges from zero to 21, with higher scores indicating a more severe level of anxiety (9).

Confirmed COVID-19 was defined as testing positive for SARS-CoV-2 (RT-PCR or antigen tests) or experiencing an episode of acute symptoms that could be considered symptomatic SARS-CoV-2 infection.

### The SEIQHCVR model

**Figure 1:**
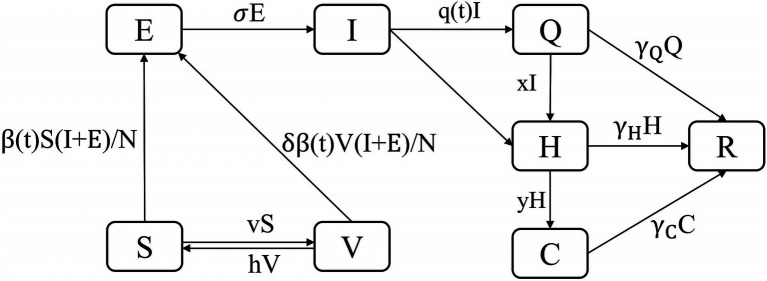
The diagram of the SEIQHCVR model.

Although the SEIR model was widely used in early studies (10, 11), considering the new situation of epidemic liberalization in China where the population chooses to stay at home after infection or those with serious conditions need to be hospitalized, the SEIR model can no longer accurately simulate and predict the epidemic trend in order to better fit the real situation of the epidemic. In this study, we constructed the SEIQHCVR model with the following set of differential equations (Figure 1). The model assumed that after exposure to infectious sources *I* and *E*, *S* will be infected with probability $\beta \left(t\right)$ and thus entered into *E*; over time, the protective efficacy of the vaccine gradually weakened, and *V* will be infected with probability $\delta \beta \left(t\right)$ and thus entered into *E*; and *E* will go through an incubation period of 1/σ and thus entered into *I*.

$$dS/dt=-\beta \left(t\right)S\left(I+E\right)/N-vS+hV$$

$$dE/dt=\beta \left(t\right)S\left(I+E\right)/N+\delta \beta \left(t\right)V\left(I+E\right)/N-\sigma E$$

$$dI/dt=\sigma E-\left(q\left(t\right)+x\right)I$$

$$dQ/dt=q\left(t\right)I-\gamma_{Q}Q$$

$$dH/dt=\mathrm{xI}-\left(y+\gamma_{H}\right)H$$

$$dC/dt=yH-\gamma_{C}C$$

$$dV/dt=vS-hV-\delta \beta \left(t\right)V\left(I+E\right)/N$$

$$dR/dt=\gamma_{Q}Q+\gamma_{H}H+\gamma_{C}C$$

The definitions of the compartments and parameters in the above equations are displayed in Table 1. In this study, $\beta \left(t\right)$ and $q\left(t\right)$ are piecewise functions that represent the time-varying transmission rate and time-varying home quarantine rate, respectively. $\beta \left(t\right)$ and $q\left(t\right)$ are expressed as:

$$\beta \left(t\right)=\{\begin{array}{cc} \beta_{0} & t<t_{1}\\ \beta_{0}\times e^{-w\left(t-t_{1}\right)} & t_{1}\leq t<t_{2}\\ \beta_{0}\times e^{-o\left(t-t_{2}\right)} & t\geq t_{2} \end{array}$$

$$q\left(t\right)=\{\begin{array}{cc} q_{0} & t<t_{1}\\ \left(q_{0}-q_{1}\right)\times e^{-r\left(t-t_{1}\right)}+q_{1} & t_{1}\leq t<t_{2}\\ q_{2} & t\geq t_{2} \end{array}$$

The Metropolis–Hastings (M–H) algorithm in the Markov Chain Monte Carlo (MCMC) method was used for parameter estimation (12), including $\beta_{0}$ , w , o , δ , $q_{1}$ and r . The values and sources of the compartments and parameters are listed in Table 2. Due to the uncertainty of the parameters, we performed sensitivity tests on these six parameters. Although $\beta_{0}$ showed higher sensitivity than the other parameters, the behavior pattern remained constant, increasing confidence to the model. The results of the sensitivity analyses are presented in Supplementary Figure S1.

**Table 1:** The definitions of the compartments and parameters.

Compartments & parameters		Definitions
*S*_0_		Susceptible individuals
*E*_0_		Exposed individuals
*I*_0_		Infected individuals
*Q*_0_		Home quarantine individuals
*H*_0_		Inpatients
*C*_0_		Critically ill patients
*V*_0_		Individuals immunized by vaccination
*R*_0_		Recovered individuals
$\beta \left(t\right)$	$\beta_{0}$	Initial transmission rate
	w	Exponential decline rate of transmission rate ( $t_{1}\leq t<t_{2}$ )
	o	Exponential decline rate of transmission rate ( $t\geq t_{2}$ )
$q\left(t\right)$	$q_{0}$	Initial home quarantine rate
	$q_{1}$	Maximum home quarantine rate after implementation of control measures
	r	Exponential growth rate of home quarantine rate
	$q_{2}$	Home quarantine rate ( $t\geq t_{2}$ )
σ		Incubation rate
x		Transition rate of infected individuals to inpatients
y		Transition rate of inpatients to critically ill patients
$\gamma_{Q}$		Recovery rate of home quarantine individuals
$\gamma_{H}$		Recovery rate of inpatients
$\gamma_{C}$		Recovery rate of critically ill patients
v		Immunity threshold (vaccination rate × vaccine protection rate)
h		Rate at which an immunized person becomes susceptible due to decreased antibody levels
δ		Coefficient of transmission rate of an immunized person becomes exposed after being infected due to decreased antibody levels

**Table 2:** The values and sources of the compartments and parameters.

Compartments & parameters		Values			Sources
		Shanxi province	Urban	Rural	
*S*_0_		34,173,734	21,383,538	12,790,196	Actual epidemic
*E*_0_		567,620	341,846	225,774	Actual epidemic
*I*_0_		174,262	106,110	68,152	Actual epidemic
*Q*_0_		0	0	0	Actual epidemic
*H*_0_		0	0	0	Actual epidemic
*C*_0_		0	0	0	Actual epidemic
*V*_0_		0	0	0	Actual epidemic
*R*_0_		0	0	0	Actual epidemic
$\beta \left(t\right)$	$\beta_{0}$	0.714 (0.711, 0.718) day^−1^	0.735 (0.730, 0.741) day^−1^	0.690 (0.681, 0.696) day^−1^	MCMC
	w	0.137 (0.134, 0.139) day^−1^	0.137 (0.135, 0.139) day^−1^	0.136 (0.131,0.139) day^−1^	MCMC
	o	0.005 (0.0047, 0.0053) day^−1^	0.004 (0.0037, 0.0042) day^−1^	0.005 (0.0046, 0.0053) day^−1^	MCMC
$q\left(t\right)$	$q_{0}$	0.1 day^−1^	0.1 day^−1^	0.1 day^−1^	Actual epidemic
	$q_{1}$	0.633 (0.628, 0.639) day^−1^	0.575 (0.563, 0.590) day^−1^	0.608 (0.596, 0.625) day^−1^	MCMC
	r	0.128 (0.120, 0.135) day^−1^	0.129 (0.123, 0.139) day^−1^	0.099 (0.087, 0.112) day^−1^	MCMC
	$q_{2}$	0.017 day^−1^	0.017 day^−1^	0.017 day^−1^	Actual epidemic
σ		1/4 day^−1^	1/4 day^−1^	1/4 day^−1^	Actual epidemic
x		0.005 day^−1^	0.005 day^−1^	0.005 day^−1^	Actual epidemic
y		0.03 day^−1^	0.03 day^−1^	0.03 day^−1^	Actual epidemic
$\gamma_{Q}$		0.14 day^−1^	0.14 day^−1^	0.14 day^−1^	Actual epidemic
$\gamma_{H}$		0.1 day^−1^	0.1 day^−1^	0.1 day^−1^	Actual epidemic
$\gamma_{C}$		0.1 day^−1^	0.1 day^−1^	0.1 day^−1^	Actual epidemic
v		0.9 day^−1^	0.9 day^−1^	0.9 day^−1^	Actual epidemic
h		0.73 day^−1^	0.73 day^−1^	0.73 day^−1^	Actual epidemic
δ		0.316 (0.312, 0.319)	0.317 (0.314, 0.319)	0.280 (0.273, 0.289)	MCMC

### Basic reproduction number ( $R_{0}$ ) and effective reproduction number ( $R_{t}$ )

The basic reproduction number estimates the mean number of secondary cases generated by a single infected individual in an entirely susceptible human population, and is primarily used to measure the infectiousness and transmission efficiency of a pathogen (13). To curb epidemics of infectious diseases and develop more rational prevention and control measures, an effective reproduction number is required to reflect the transmission power of infectious diseases in the population in real time. The next-generation matrix method is widely used in the process of solving $R_{0}$ and $R_{t}$ (14). $R_{0}$ and $R_{t}$ can be expressed as the spectral radius of the next-generation matrix through derivation of the local stability of the disease-free equilibrium point (15). From the constructed transmission dynamics model, there are two categories of populations with infectivity, namely, *E* and *I*. For a disease-free equilibrium solution, *E* = *I* = 0.

$F_{i}$ represents new infection terms, and the nonsingular matrix, $V_{i}$ denotes the remaining transfer terms, which can be described as follows (16):

$$F_{i}=\left[\begin{array}{c} \frac{\beta \left(t\right)S\left(I+E\right)}{N}+\frac{\delta \beta \left(t\right)V\left(I+E\right)}{N}\\ 0 \end{array}\right],V_{i}=\left[\begin{array}{c} \sigma E\\ -\sigma E+q\left(t\right)I+\mathrm{xI} \end{array}\right]$$

Partial derivatives with respect to *E* and *I* results to:

$$F=\left[\begin{array}{cc} \frac{\beta \left(t\right)S}{N}+\frac{\delta \beta \left(t\right)V}{N} & \frac{\beta \left(t\right)S}{N}+\frac{\delta \beta \left(t\right)V}{N}\\ 0 & 0 \end{array}\right],\;V=\left[\begin{array}{cc} \sigma & 0\\ -\sigma & q\left(t\right)+x \end{array}\right]$$

The product of two matrices ( F and $V^{-1}$ ) gives:

$$\begin{array}{l} FV^{-1}=[\begin{array}{c} \frac{\beta \left(t\right)S/N+\delta \beta \left(t\right)V/N}{q\left(t\right)+x}+\frac{\beta \left(t\right)S/N+\delta \beta \left(t\right)V/N}{\sigma }\\ 0 \end{array}\\ \;\begin{array}{c} \frac{\beta \left(t\right)S/N+\delta \beta \left(t\right)V/N}{q\left(t\right)+x}\\ 0 \end{array}]\; \end{array}$$

The spectral radius of $\rho \left(FV^{-1}\right)$ , gives the effective reproduction number is given by:

$$\begin{array}{l} R_{t}=\rho \left(FV^{-1}\right)=\frac{\beta \left(t\right)S/N+\delta \beta \left(t\right)V/N}{q\left(t\right)+x}\\ \;+\frac{\beta \left(t\right)S/N+\delta \beta \left(t\right)V/N}{\sigma } \end{array}$$

## Results

### Socio-demographic characteristics and infection status of the respondents

A total of 998,409 valid questionnaires were collected, of which 384,052 (38.47%) were male, 568,372 (56.93%) were aged 20–59 years and 590,826 (59.18%) were from urban areas. Overall, 851,464 respondents were affected by the novel coronavirus, with an infection rate of 85.28% (851,464/998,409). Fever (74.90%) and dry cough (63.32%) are the most common and typical symptoms. Respondents who received four or more doses of COVID-19 vaccine had a lower rate of infection, and the shorter the time since the last vaccination, the lower the rate of infection.

### Temporal distribution of novel coronavirus infections in Shanxi province

**Figure 2:**
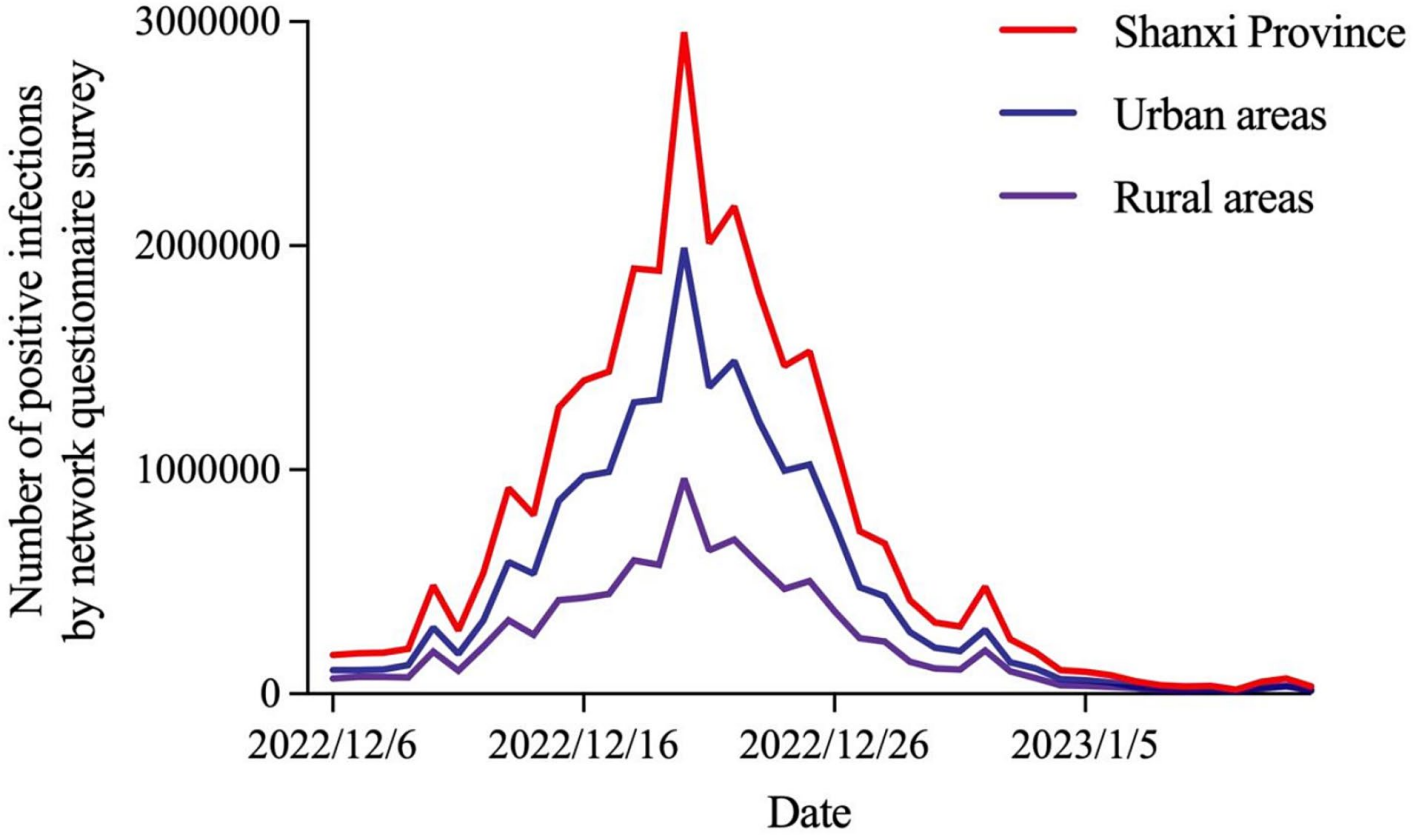
Trends of novel coronavirus infections in Shanxi province.

The current outbreak in Shanxi province has developed rapidly since early December 2022, and the daily number of new infections in 1 day showed an obvious downward trend after reaching a peak on December 20, 2022. The infection trends of urban and rural residents were similar to those of the entire province (Figure 2).

### Prediction of positive infections in Shanxi province

**Figure 3:**
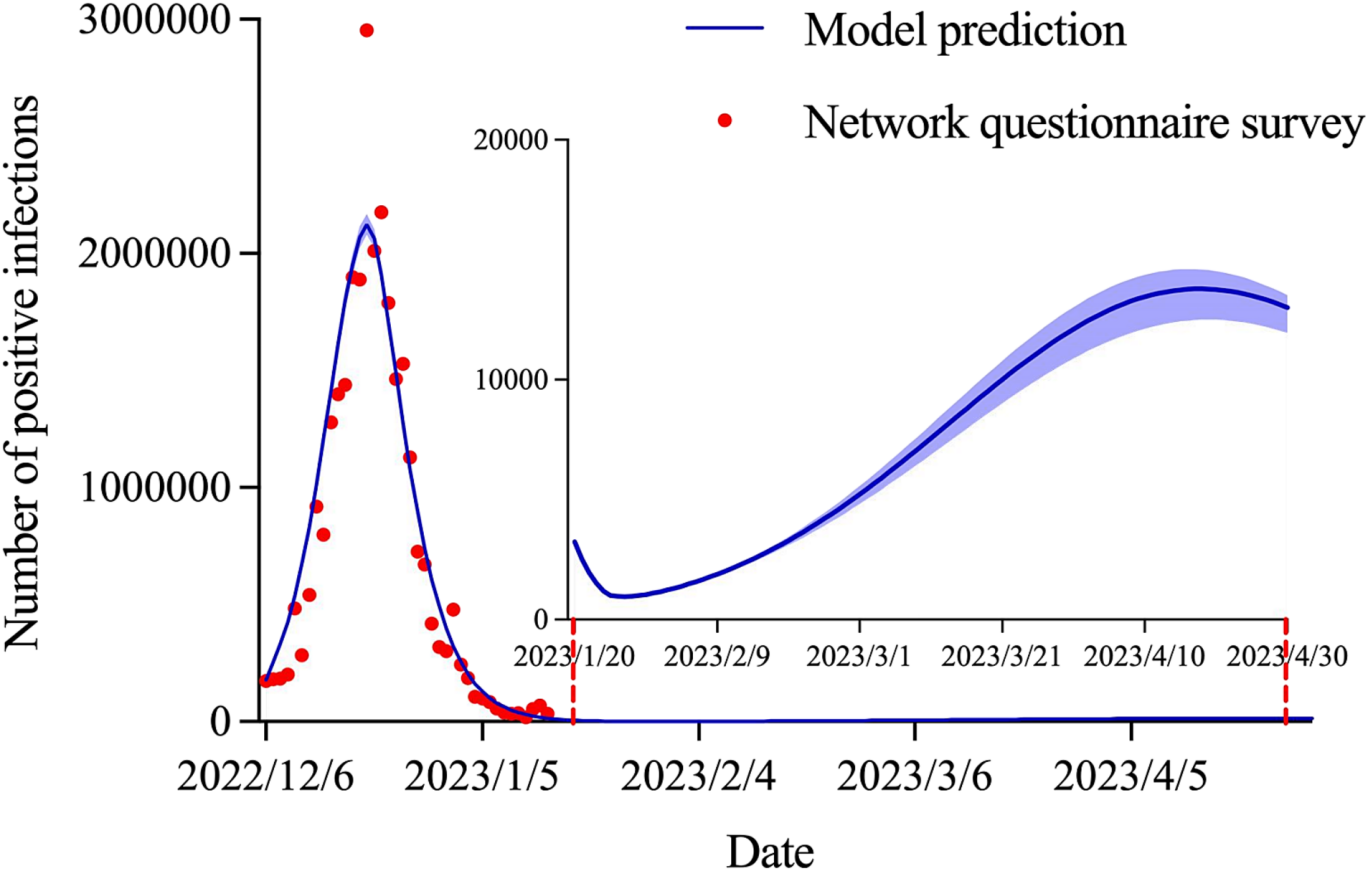
Estimation and prediction of the daily number of positive infections in Shanxi province.

As shown in Figure 3, the number of positive infections peaked at 2,124,312 (95% CI: 2,081,372–2,167,756) on December 20, 2022 and then decreased continuously to 14,458 (95% CI: 14,151–14,852) on January 14, 2023, with a decrease of 99.32%. The outbreak subsided in late January, with an epidemic size of 30,629,499 (95% CI: 30,020,725–31,277,435). The coefficient of determination ( $R^{2}$ ) was 0.925. In addition, a small wave of COVID-19 infections may re-emerge at the end of April.

### Prediction of positive infections in urban and rural areas

**Figure 4:**
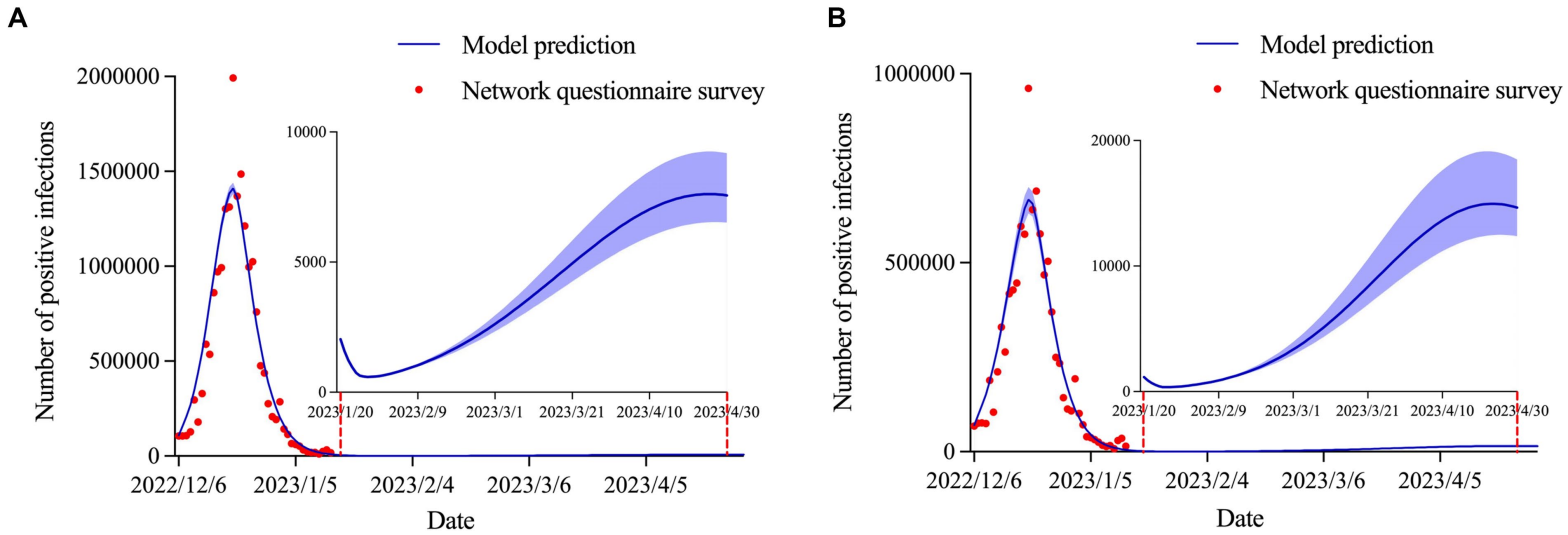
Estimation and prediction of the daily number of positive infections in **(A)** urban areas and **(B)** rural areas.

We predicted that the positive infections in urban ( $R^{2}$ = 0.924) and rural ( $R^{2}$ = 0.917) areas would reach inflection points on December 20, 2022, with 1,410,406 (95% CI: 1,380,093–1,440,164) and 666,908 (95% CI: 630,702–700,145) cases, respectively. According to data up to January 14, 2023, the infection rate in urban areas will be approximately 16% higher than that in rural areas (92.07% vs. 76.08%). By late January, the cumulative number of infections in urban and rural areas was predicted to reach 20,134,362 (95% CI: 19,699,686–20,558,869) and 9,973,386 (95% CI: 9,463,883–10,465,840), respectively. Similarly, a small wave of COVID-19 infections may re-emerge at the end of April in both urban and rural areas (Figure 4).

### Prediction of inpatients and critically ill patients

**Figure 5:**
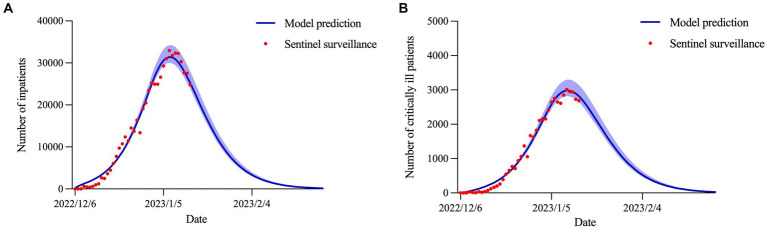
Estimation and prediction of the daily number of **(A)** inpatients and **(B)** critically ill patients.

The model predicted that the number of inpatients ( $R^{2}$ = 0.984) and critically ill patients ( $R^{2}$ = 0.988) would peak on January 7, 2023 and January 10, 2023, with the number of peak cases of 31,426 (95% CI: 30,048–34,258) and 2,987 (95% CI: 2,820–3,298), respectively. The number of inpatients and critically ill patients was expected to be close to its end in early February (Figure 5).

### Prediction of effective reproduction number

**Figure 6:**
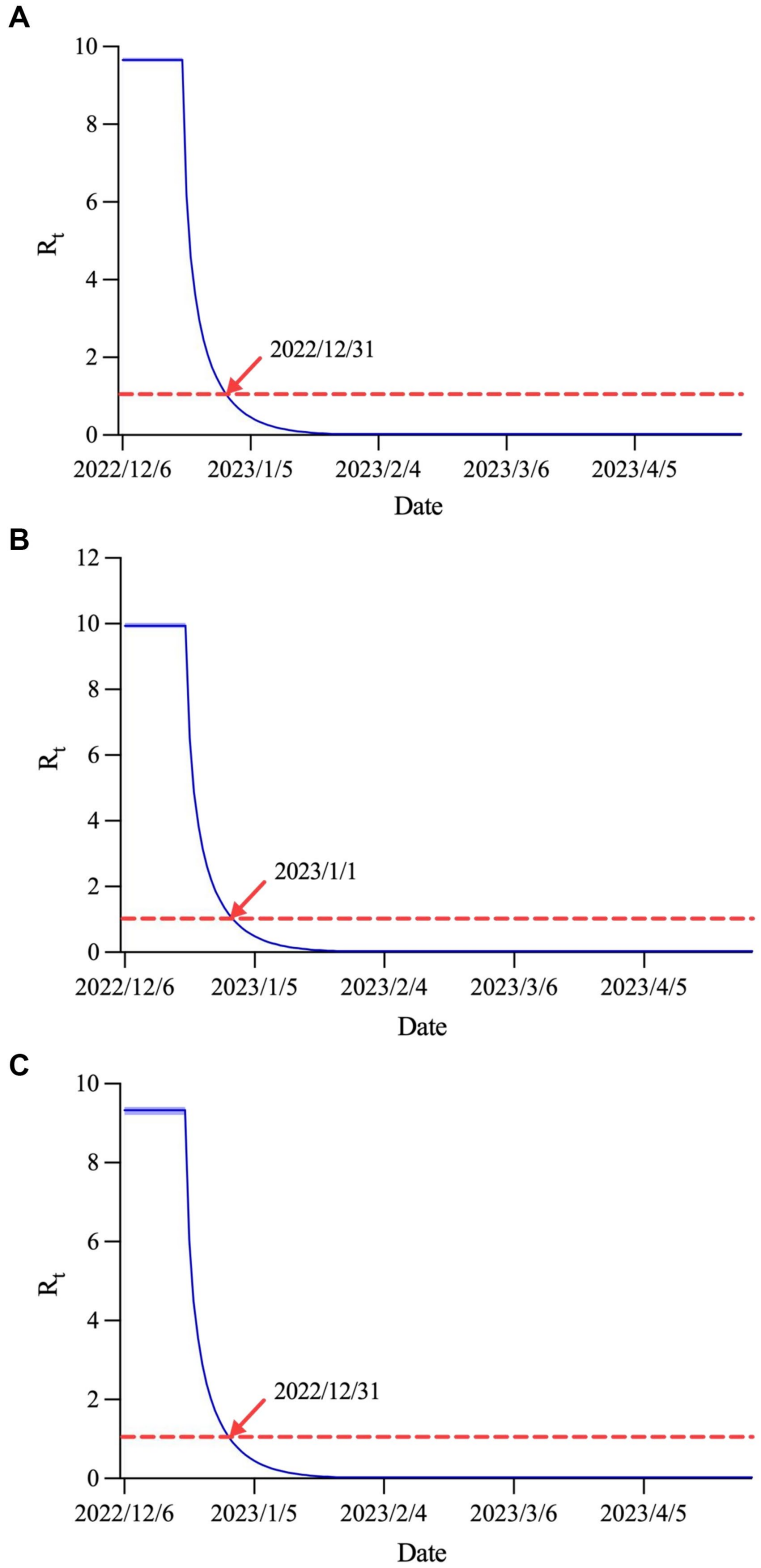
$R_{t}$ curves in **(A)** Shanxi province, **(B)** urban areas and **(C)** rural areas.

Figure 6 displays the estimated $R_{t}$ curves in Shanxi province and urban and rural areas. The values of $R_{0}$ were 9.66 (95% CI: 9.62–9.71), 9.94 (95% CI: 9.87–10.02) and 9.33 (95% CI: 9.21–9.41), respectively. Subsequently, $R_{t}$ began to decline rapidly until it dropped below 1.0 on December 31, 2022 in Shanxi province and rural areas, and below 1.0 on January 1, 2023 in urban areas.

## Discussion

Coronaviruses are bound to exist for a long time in nature, with a considerable decline in pathogenicity from the early stage, as the diseases caused by them will gradually evolve into common respiratory infectious diseases, considering that Omicron has become the dominant strain worldwide.

China has downgraded the coronavirus management level to Class B infectious disease and cancelled the quarantine requirements for international arrivals in accordance with the Frontier Health and Quarantine Law of the People’s Republic of China. Following this adjustment, China’s COVID-19 prevention and control efforts will focus on protecting health and preventing severe cases. Measures will be rolled out to protect people’s lives and health, and minimize the impact of the epidemic on economic and social development. When large-scale testing and identification of all cases became impossible after the liberalization of epidemic prevention, our study intended to estimate infection prevalence in Shanxi province, located in northern China, through a network questionnaire survey combined with sentinel surveillance, to adjust the allocation of medical resources in due course and ensure evidence-based policy-making. The recent wave of novel coronavirus infection in Shanxi province has passed its peak and continues to decline, with infections in urban and rural areas largely synchronized with Shanxi province. The number of inpatients and critically ill patients is also currently in a steady decline. During the Spring Festival, the spread of the novel coronavirus did not rebound significantly. However, a small wave of COVID-19 infections may re-emerge at the end of April.

Since the outbreak of COVID-19, many studies have made prospective predictions of infections, deaths, and hospitalizations using the infectious disease dynamics model, which is considered to be an important tool for forecasting the prevalence of COVID-19 (17–19). In previous studies, we have utilized a modified SEIR model to successfully predict the number of COVID-19 cases in Shanghai and Hohhot (20, 21). In this study, we constructed a dynamics model of SEIQHCVR in line with a “Class B infectious disease Class B management” control strategy. Within the framework of the classical SEIR model, we further added four compartments: home quarantine individuals, inpatients, critically ill patients, and individuals immunized by vaccination; at the same time, we took into account the significance of non-conventional parameters, such as the maximum home quarantine rate after implementation of control measures, so that the predictions of the SEIQHCVR model were in good agreement with the actual situation. We have reason to believe that this model will play an important role in predicting the transmission dynamics of novel coronavirus infection after the opening of the epidemic in China.

By deducing the trends of positive infections, inpatients, and critically ill patients in Shanxi province, three important conclusions were drawn. In terms of the epidemic peak, the number of infections in Shanxi province, as well as in urban and rural areas, peaked on December 20, 2022, with the peak of inpatients and critically ill patients occurring 2 to 3 weeks after the peak of infections. In terms of the final affected population, as of January 31, 2023, the final infection rate in urban areas (92.23%) was higher than that in the Shanxi province (87.72%) and rural areas (76.23%). In terms of $R_{0}$ and $R_{t}$ , the values of $R_{0}$ were, in descending order, urban areas (9.94), Shanxi province (9.66) and rural areas (9.33), and the $R_{t}$ values of all three were lower than 1.0 in less than a month. The infection rate in this round of the epidemic in Shanxi province is almost the same as that in economically developed areas such as Beijing and Shanghai. For example, the cumulative infection attack rate in Beijing was 92.3% (95% CI: 91.4–93.1) on January 31, 2023 and 80%–85% of Shanghai residents would be infected in early January (22, 23). However, by November 9, 2022, 94% of the US population were estimated to have been infected at least once (24). We speculated that this may be because the epidemic was liberalized much earlier in the United States than in China.

After an in-depth analysis of the above findings, we found that the trend of infection was generally consistent between urban and rural areas, with no later inflection point in rural areas than in urban areas. This suggests that rural areas also need to be prepared in advance to accelerate their capacity to receive and treat novel coronavirus infections. In addition, 2 to 3 weeks following the peak of infection is the prime time to deploy emergency medical treatment resources. Specialized medical staff and intensive care units should be expanded to cope with the treatment of inpatients and critically ill patients. Another point of concern is that there is a possibility of a small wave of COVID-19 infections in late April 2023. It is recommended that the grass-roots governments in urban and rural areas should make a good stockpile of anti-epidemic materials, such as antigen detection kits and disinfection supplies, well in advance. Furthermore, the focus of future prevention efforts should be to ramp up the full vaccination and booster vaccination rates among those at high risk of severe illness, such as the older adults aged 60 years and above, to mitigate the possible harm caused by the second round of infection.

Articles published in the world-leading medical journal The Lancet pointed out that the Chinese government has saved thousands of lives through its huge investment in public health, and that country’s experience in fighting the COVID-19 pandemic is worth learning by other countries (25). Over the past 3 years, China has maintained its strategic initiative in its fight against COVID-19, and has been optimising and adjusting its COVID-19 prevention and control measures in light of the evolving situation. However, China has entered a new stage in the COVID-19 response. Various regions and authorities in the country increased their efforts during the transition period. The COVID-19 vaccination rate in the older adults should be increased, and preparations for drugs and test reagents should also be improved. Moreover, China should increase spending on medical resources, adjust quarantine areas, and implement other preventive measures for the treatment and prevention of the virus.

Our study has several limitations. First, six parameters were estimated using the MCMC method and therefore inevitably subject to a certain deviation. In the future, there is a need for further data collection to analyze these parameters. Second, the SEIQHCVR model we constructed assumed that the total population is constant throughout the entire process, whereas in the real world, the total population is always changing, which indicates that inter-regional human mobility was not taken into account in the model. Third, the under-representation of children and older people in the network questionnaire survey may have introduced some information bias. Finally, when filling out the questionnaire, the self-reported infection status with fever as the main symptom or antigen test results may also be biased.

## Conclusion

The current outbreak in Shanxi province is now at a low prevalence level and has generally passed the peak of infection, hospitalization and critical illness. Given the continued mutation of the Omicron variant and dwindling levels of antibodies in the population, it is reasonable to speculate that multiple waves of Omicron outbreaks may occur in mainland China in the future. China also kept its rates of infection and mortality at the world’s lowest level. As China’s COVID-19 response has entered a new stage, it continues to optimize its science-based COVID-19 prevention and control measures to ensure smooth transition and maintain social stability. To safeguard people’s lives and health to the greatest extent possible and to minimize the impact of COVID-19 on economic and social development, it is necessary to make full use of the advantages of big data, artificial intelligence, and mathematical models to scientifically predict the development trend of the epidemic and provide timely technical support for prevention and control in various regions.

## Data availability statement

The original contributions presented in the study are included in the article/Supplementary material, further inquiries can be directed to the corresponding authors.

## Ethics statement

This study was approved by the Ethics Committee of Shanxi Medical University. The studies were conducted in accordance with the local legislation and institutional requirements. Written informed consent for participation in this study was provided by the participants’ legal guardians/next of kin. Informed consent was obtained through the online survey, and participants remained anonymous.

## Author contributions

YM: Funding acquisition, Methodology, Software, Validation, Writing – original draft. SX: Methodology, Software, Visualization, Writing – review & editing. YL: Methodology, Software, Writing – review & editing. JP: Methodology, Software, Validation, Writing – review & editing. JG: Methodology, Software, Validation, Writing – review & editing. AD: Data curation, Investigation, Resources, Writing – review & editing. ZX: Data curation, Investigation, Resources, Writing – review & editing. JL: Data curation, Formal analysis, Project administration, Writing – review & editing. LL: Data curation, Formal analysis, Project administration, Writing – review & editing. LH: Data curation, Formal analysis, Project administration, Writing – review & editing. TW: Data curation, Formal analysis, Funding acquisition, Project administration, Writing – review & editing. HY: Conceptualization, Supervision, Writing – review & editing. JX: Conceptualization, Funding acquisition, Supervision, Writing – review & editing.

## Supplementary material

SUPPLEMENTARY FIGURE S1 Sensitivity tests of six parameters estimated using the MCMC method.
