# Supplementary material for: Predicting the transmission dynamics of novel coronavirus infection in Shanxi province after the implementation of the “Class B infectious disease Class B management” policy
Source: Front Public Health. 2023 Dec 22;11:1322430. doi: 10.3389/fpubh.2023.1322430 (PMC10768892; doi:10.3389/fpubh.2023.1322430)
Supplement: SUPPLEMENTARY FIGURE S1 — Sensitivity tests of six parameters estimated using the MCMC method. [file Image_1.pdf]

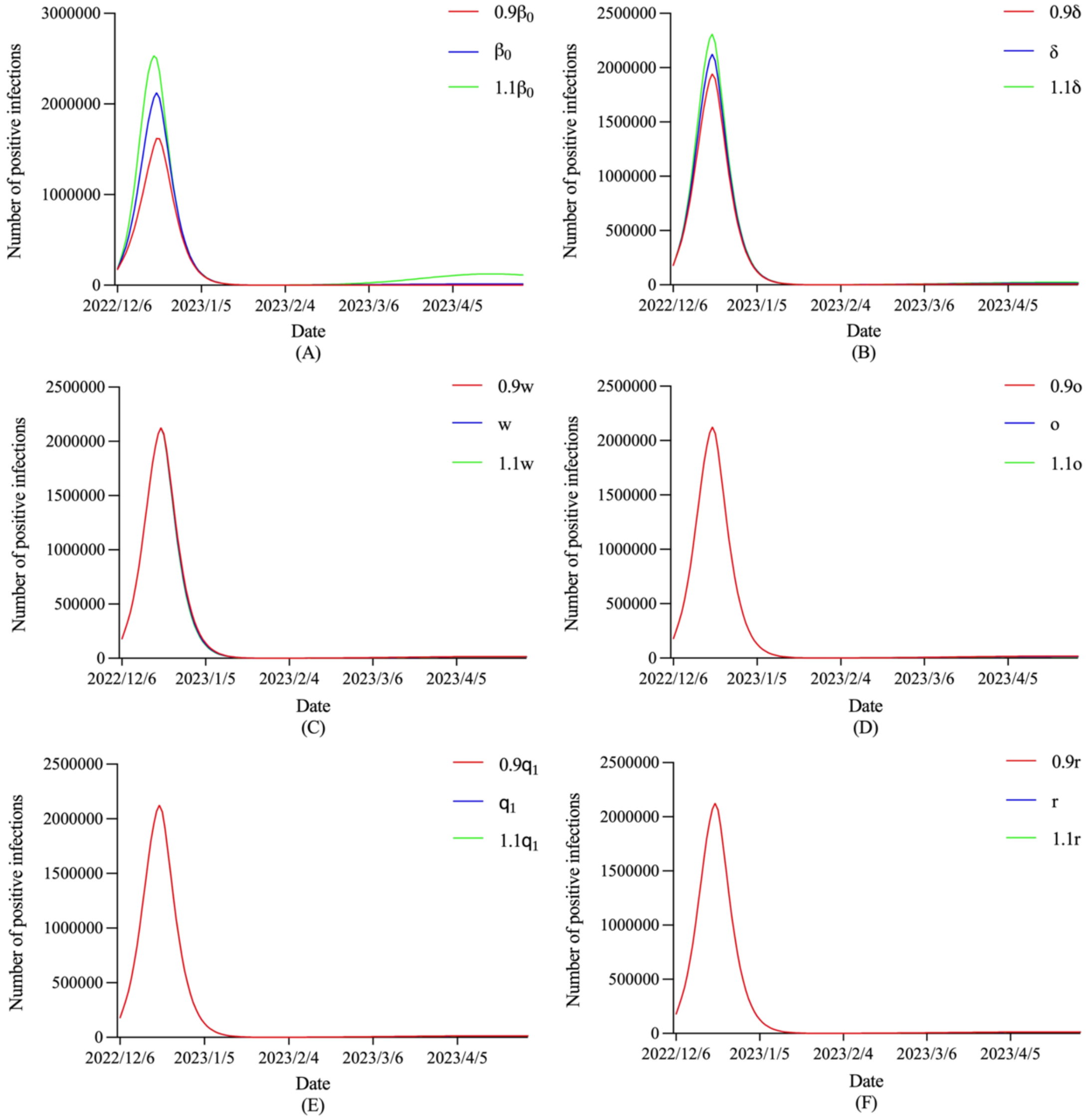

FIGURE S1 Sensitivity tests for six parameters. (A) β_0, (B) δ, (C) w, (D) o, (E) q_1 and (F) r.

The initial transmission rate (β_0) had high sensitivity. 10% increase or decrease caused the simulation results to vary over a wide range. However, the behavior pattern remained constant.

The coefficient of transmission rate of an immunized person becomes exposed after being infected due to decreased antibody levels (δ) had little sensitivity over model behavior. 10% increase or decrease generated only slight increase or decrease in the simulation results.

The exponential decline rate of transmission rate ($t_1 \leq t < t_2$) (w), exponential decline rate of transmission rate ($t \geq t_2$) (o), maximum home quarantine rate after implementation of control measures (q_1), and exponential growth rate of home quarantine rate (r) had almost no sensitivity over model behavior. 10% increase or decrease almost had no impact on the simulation results.
